# A Case of Chronic Cough and Pneumonia Secondary to a Foreign Body

**DOI:** 10.1155/2017/3092623

**Published:** 2017-09-18

**Authors:** Joan Dabu, Meredith Lindner, Moh'd Azzam, Anas Al-Khateeb, Muqueet Kadri, Sharath Bellary, Richard Miller

**Affiliations:** New York Medical College, St. Michael's Medical Center, Pulmonary Department, Newark, NJ, USA

## Abstract

Foreign body aspiration occurs when a solid or semisolid object becomes lodged in the larynx or trachea. It can be a life-threatening emergency, especially if it is large enough to occlude the airway. However, small aspirated objects may go unnoticed until symptoms occur. Therefore, it is frequently misdiagnosed. A high level of clinical suspicion, patient's risk factors, and thorough history and physical examination are essential in making the diagnosis. It should be considered in cases where there is unresolved chronic cough with or without associated recurrent pneumonia especially in patients with risks for aspiration.

## 1. Introduction

Foreign body aspiration is infrequently seen in adults. A foreign body is commonly lodged in the right, especially in the right intermediate bronchus. Precipitating factors include CNS dysfunction, trauma intubation, dental procedure, and pulmonary disease [1]. Symptoms include shortness of breath, fever, and hemoptysis. In this case, it presents as chronic cough with recurrent pneumonia. Bronchoscopy is recommended as initial management for diagnosis and possible treatment.

## 2. Case Description

This is the case of a 55-year-old African American female, with past medical history of hypertension and illicit drug use, who presented with worsening productive cough. The patient stated that the cough had been ongoing for the past 5 months but had worsened in severity over the last 2 weeks prior to admission. She complained of associated fever and chills for two days, sore throat, and copious white sputum. She denied shortness of breath, wheezing, and pleuritic chest pain. She also denied having hemoptysis. Patient denied having had any recent exposure to sick individuals. She is a 40-pack-year smoker, drinks alcohol socially, and admits drug use.

Upon admission temperature was 98.1 F, blood pressure 112/61, heart rate 92 beats per minute, respiratory rate 18 breaths per minute, and saturation 99% in room air. Physical examination was significant for left lower lobe rhonchi without wheezing or rales. Laboratory studies were significant for elevated leukocyte count of 13.0 × 10^3^/*μ*L with a negative procalcitonin level. All other laboratory values were within normal limits. Urine drug screen was positive for opiates.

**Figure 1:**
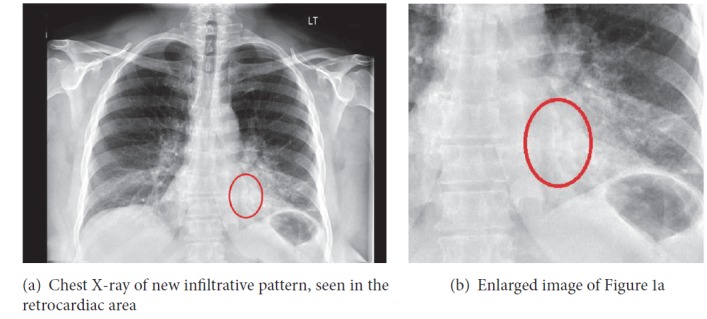


**Figure 2:**
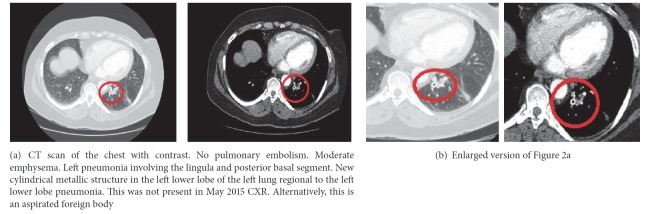


Chest X-ray revealed left-sided infiltrative pattern (Figures 1(a) and 1(b)). A CT scan of the chest (Figures 2(a) and 2(b)) showed left-sided pneumonia involving the lingula and posterior basal segment and endobronchial occlusion of the posterior segment of the left lower lobe, possibly foreign body, not present on prior chest X-ray. Patient is subsequently scheduled for bronchoscopy.

**Figure 3:**
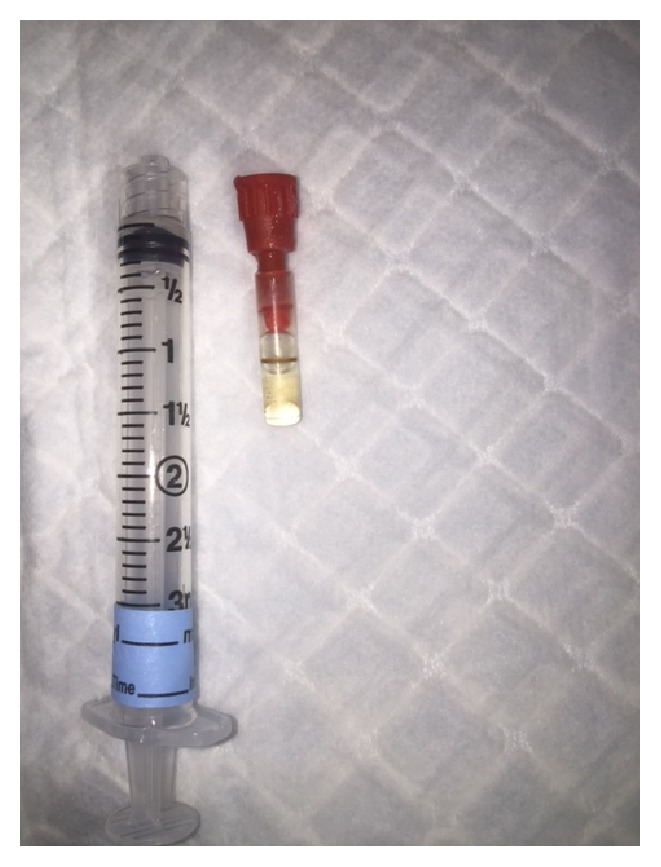
Extracted foreign body, removed with biopsy forceps during bronchoscopy.

Prior to bronchoscopy, the patient disclosed additional information about her social history. She stated that 5 months prior to the admission, she was abusing cocaine and during an altercation with law enforcement she attempted to ingest a vial of cocaine. Bronchoscopy was performed, which revealed a significant circumferential inflammation of the left lower lobe segment and a foreign body noted in the postbasal left lower lobe bronchus. The foreign body was successfully removed with biopsy forceps, revealing a synthetic vial measuring 3.2 × 0.7 × 0.7 cm (Figure 3).

Following bronchoscopy, the patient's cough improved significantly. She denied having shortness of breath, wheezing, and fever. Patient was sent home with oral antibiotics along with her home medications.

## 3. Discussion

Foreign body aspiration is more common in children than in adults. Predisposing factors are frequently associated with foreign body aspiration in adults, particularly, altered mental status, neuromuscular disease, head trauma, and psychiatric disorders [1]. Impaired cough reflex and dysphagia contribute to the pathology of aspiration [2]. Foreign bodies may result in serious complications if left undiagnosed. This includes pneumonia, lung abscess, atelectasis, and bronchiectasis [3]. Endobronchial foreign body is one of the suspected causes of persistent obstructive pneumonia [4]. Signs and symptoms are nonspecific which include cough, shortness of breath, wheezing, and/or hemoptysis [2].

A chest X-ray is the initial standard work-up for a suspected foreign body aspiration. The most common location for a foreign body is the right bronchus, especially the right lower lobe, due to its vertical orientation [5]. Signs of atelectasis and/or chronic or recurrent infiltrate in chest imaging may raise suspicion for an endobronchial lesion or foreign body. However, a normal chest radiograph is not uncommonly seen [2]. Therefore, a high index of clinical suspicion is at times necessary to make the diagnosis, regardless of an unremarkable chest radiograph. A computed tomography (CT) scan of the chest is indicated with a negative chest X-ray and a high clinical suspicion.

Gustav Killian performed the first bronchoscopy in 1897 to diagnose, evaluate, and treat foreign body aspiration [2]. Since then, bronchoscopy has been the gold standard in diagnosing foreign body aspiration in adults. It is used without added risk to patients due to a more invasive procedure such as surgery. A variety of accessories, which include specially designed forceps, snares, baskets, balloons, and magnets, are used with the bronchoscopy for the extraction of a foreign body [6]. Cryotherapy, electrocautery, or laser may be necessary to remove granulation tissues that have formed over a longer period of time [6].

## 4. Conclusion

Foreign bodies may result in serious complications if left undiagnosed. In appropriate clinical settings, foreign body aspiration should be considered in patients with recurrent pneumonia and persistent cough. In addition, it can also result in lung abscess, atelectasis, and bronchiectasis. A radiographic image is the initial standard work-up for a suspected foreign body aspiration [7]. Fiber-optic bronchoscopy is the preferred tool in diagnosing foreign body in adults. It can also be used in an attempt to remove the foreign body without added risk to patients due to a more invasive procedure such as surgery.
